# Effect of Magnetic Anisotropy and Gradient‐Induced Dzyaloshinskii‐Moriya Interaction on the Formation of Magnetic Skyrmions

**DOI:** 10.1002/smll.202505204

**Published:** 2025-07-26

**Authors:** Adam Erickson, Qihan Zhang, Hamed Vakili, Edward Schwartz, Suvechhya Lamichhane, Chaozhong Li, Boyu Li, Dongsheng Song, Guozhi Chai, Sy‐Hwang Liou, Alexey A. Kovalev, Jingsheng Chen, Abdelghani Laraoui

**Affiliations:** ^1^ Department of Mechanical & Materials Engineering University of Nebraska‐Lincoln 900 N 16th Street, W342 NH Lincoln NE 68588 USA; ^2^ Department of Materials Science and Engineering National University of Singapore Block E2, #05‐19, 5 Engineering Drive 2 Singapore 117579 Singapore; ^3^ Department of Physics and Astronomy and the Nebraska Center for Materials and Nanoscience University of Nebraska‐Lincoln 855 N 16th St Lincoln NE 68588 USA; ^4^ Key Laboratory for Magnetism and Magnetic Materials of Ministry of Education, School of Physical Science and Technology Lanzhou University Lanzhou 730000 China; ^5^ Institutes of Physical Science and Information Technology Anhui University Hefei 230601 China; ^6^ National University of Singapore (Suzhou) Research Institute Suzhou Jiangsu 215123 China

**Keywords:** CoPt, Dzyaloshinskii‐Moriya interaction, magnetic anisotropy, skyrmion, topological Hall effect

## Abstract

Topological spin textures (e.g., skyrmions) can be stabilized by interfacial Dzyaloshinskii‐Moriya interaction (DMI) in the magnetic multilayer, which has been intensively studied. Recently, Bloch‐type magnetic skyrmions stabilized by composition gradient‐induced DMI (g‐DMI) have been observed in 10‐nm thick CoPt single layer. However, magnetic anisotropy in gradient‐composition engineered CoPt (g‐CoPt) films is highly sensitive to both the relative Co/Pt composition and the film thickness, leading to a complex interplay with g‐DMI. The stability of skyrmions under the combined influence of magnetic anisotropy and g‐DMI is crucial yet remains poorly understood. Here, we condcut a systematic study on the characteristics of magnetic skyrmions as a function of gradient polarity and effective gradient (defined as gradient/thickness) in g‐CoPt single layers (thickness of 10–30 nm) using magnetic force microscopy (MFM), bulk magnetometry, and topological Hall effect measurements. Brillouin light scattering spectroscopy confirms that both the sign and magnitude of g‐DMI depend on the polarity and amplitude of the composition gradient in g‐CoPt films. MFM reveals that skyrmion size and density vary with g‐CoPt film thickness, gradient polarity, and applied magnetic field. An increased skyrmion density is observed in samples exhibiting higher magnetic anisotropy, in agreement with micromagnetic simulations and energy barrier calculations.

## Graphical abstract

Magnetic force microscopy of gradient‐induced CoPt films reveals that magnetic skyrmion size and density vary with film thickness, gradient polarity, and applied magnetic field. An increased skyrmion density is observed in films exhibiting higher magnetic anisotropy. These findings show the effect of magnetic anisotropy and gradient‐induced Dzyaloshinskii‐Moriya interaction on skyrmions size and density, hh crucial for advancing high‐density magnetic memory applications.

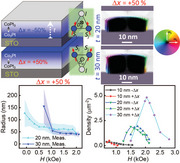

## 1 Introduction

Magnetic skyrmions are topologically protected, nanoscale spin textures that have garnered considerable interest as promising memory elements in next generation ultra‐dense and energy‐efficient spintronic devices.^[^ ^1^, ^2^ ^]^ These spin configurations are stabilized by the Dzyaloshinskii–Moriya interaction (DMI) which arises in magnetic systems with strong spin–orbit coupling (SOC) and broken inversion symmetry.^[^ ^3^, ^4^ ^]^ Interfacial DMI was shown to stabilize Néel‐type skyrmions in ultrathin ferromagnetic (FM) films interfaced with heavy metals (HM), which have been observed first at low temperatures in epitaxially grown Fe and PdFe magnetic layers on Ir,^[^ ^5^, ^6^ ^]^ and at later at room temperature in stacks of layers composed of <1‐nm‐thick Co layers sandwiched between HM layers Ir, Pt, and W.^[^ ^7^, ^8^, ^9^, ^10^ ^]^ By varying the FM and/or HM layer compositions, small size (< 50 nm) and high density (≈60 skyrmion/µm^2^) skyrmions was achieved.^[^ ^10^ ^]^ Pt/Co/Ta heterostructures with Pt‐Co alloy interlayers featuring compositional gradients were also recently studied, finding that broken inversion symmetry of graded interfaces combines interfacial and bulk‐like DMI contributions that depend on the alloy composition.^[^ ^11^ ^]^ While skyrmion properties are highly sensitive to film thickness and interfacial quality, degradation of these factors can compromise the uniformity and reliability of skyrmion behavior. Magnetic skyrmions have also been observed in chiral B20 compounds that possess bulk DMI such as MnSi,^[^ ^12^, ^13^ ^]^ Fe_1−_ *_x_* Co*_x_* Si,^[^ ^14^ ^]^ and FeGe.^[^ ^15^, ^16^ ^]^ Bulk DMI‐induced skyrmions offer potential for thermodynamic stability in bulk (thick) FM films at room temperature. However, they also come with challenges, such as the need for specific crystal structures or potential difficulties in large‐scale synthesis. Furthermore, as techniques which aim to actively manipulate DMI strength through modulation of gate voltages,^[^ ^17^, ^18^ ^]^ strain,^[^ ^19^, ^20^ ^]^ spin current,^[^ ^21^ ^]^ temperature,^[^ ^22^ ^]^ and magnetic field orientation^[^ ^23^ ^]^ are being developed, additional material geometries/properties may be useful.

Recently, gradient‐induced bulk DMI (g‐DMI) could be stabilized by the synergistic action of SOC and composition gradient‐induced bulk magnetic asymmetry materials.^[^ ^24^, ^25^ ^]^ Sizeable g‐DMI amplitudes were measured in gradient composition Co_x_Pt_1‐x_ (g‐CoPt) single‐layer systems with positive and negative composition gradient Δ*x*/*t* (*t* is the thickness of the film), where Δ*x* (up to 50%) is defined as the difference in Co percentage between the bottom and top interface.^[^ ^25^ ^]^ Such an approach opens new possibilities for engineering skyrmions with enhanced tunability and spatially varying properties. By using scanning probe based nitrogen vacancy (NV) magnetometry^[^ ^26^, ^27^, ^28^ ^]^ and magnetic force microscopy (MFM),^[^ ^29^ ^]^ we recently imaged Bloch‐type skyrmions (size ≈120–480 nm) in Δ*x* = ±50% 10 nm g‐CoPt single layers.^[^ ^30^ ^]^ The magnetic anisotropy in g‐CoPt is highly sensitive to both the relative Co/Pt composition and the film thickness, leading to a complex relationship with g‐DMI,^[^ ^24^, ^25^, ^30^ ^]^ which in turn may affect the properties of magnetic skyrmions. Therefore, elucidating the effect of magnetic anisotropy and g‐DMI on skyrmion size and density is crucial for advancing high‐density magnetic memory applications.^[^ ^1^, ^2^ ^]^

We prepared a series of g‐CoPt single layers (thickness of 10 – 30 nm) with varying effective gradients (g‐DMI), accompanied by corresponding changes in magnetic anisotropy. The reversal of the gradient polarity results in the opposite sign of the Dzyaloshinskii‐Moriya vector, *D_ijl_*, confirmed by Brillouin light scattering (BLS) spectroscopy measurements. To study the effect of magnetic anisotropy and g‐DMI on properties of skyrmions, we conducted a systematic study on the effective gradient dependent skyrmions characteristics by using MFM, bulk magnetometry, and topological Hall effect measurements. An asymmetry in the statistical nucleation of skyrmions from the uniform and polydomain starting configurations was observed. Then, an increased skyrmion density was observed in films exhibiting higher magnetic anisotropy, consistent with micromagnetic simulations of skyrmion relaxation and energy barrier calculations. These results reveal that dipolar interactions have an important role in the stabilization of skyrmions in thicker g‐CoPt films and g‐DMI primarily determines their helicity.

## 2 Results and Discussion

### 2.1 Structural and Magnetic Characterization of g‐CoPt Films

**Figure 1:**
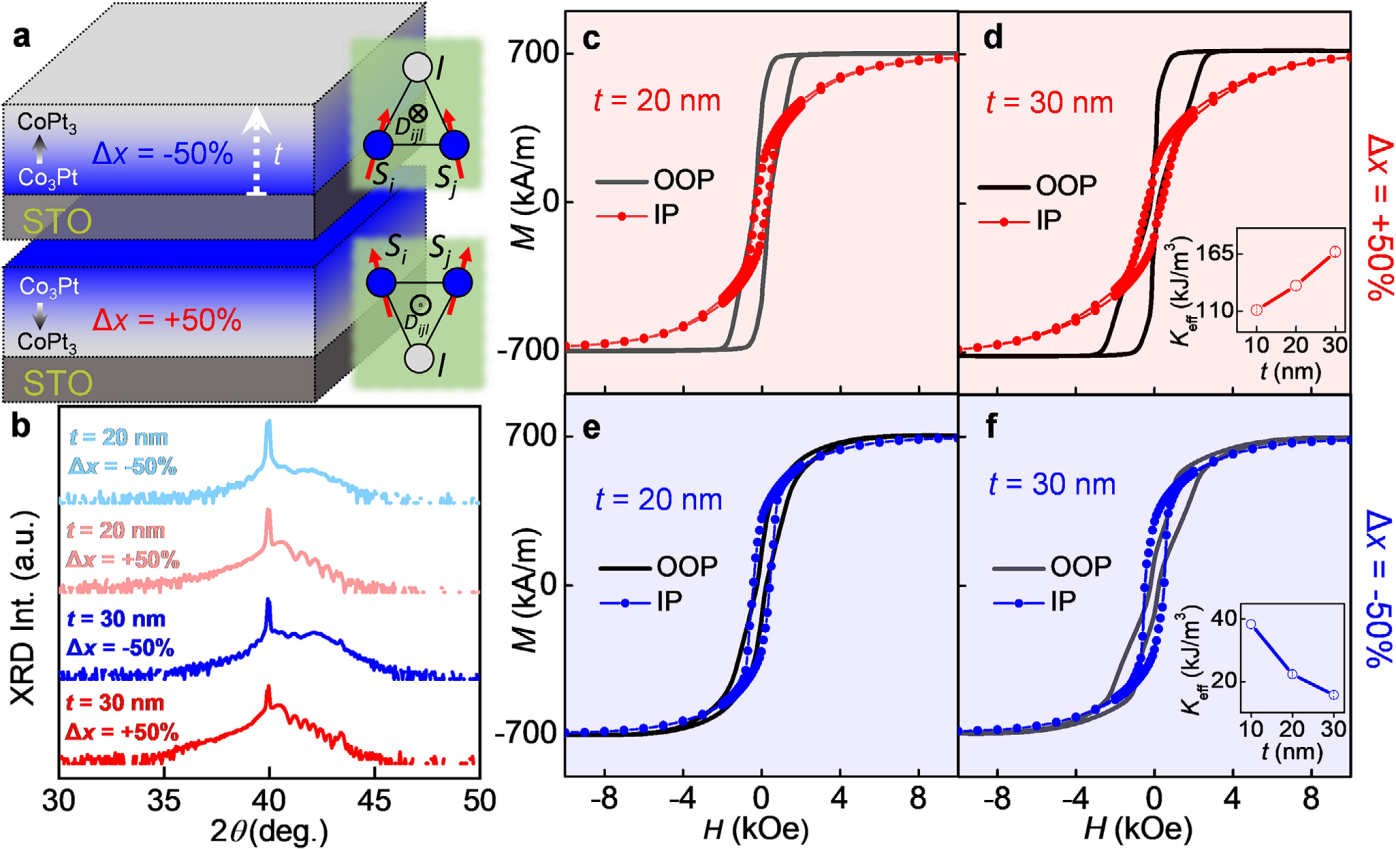
Structural and magnetic characterization of g‐CoPt films. a) Cartoon depiction of the sample geometry, indicating the defined gradient parameter, Δ*x*, for negatively and positively graded CoPt films. The statistically averaged asymmetric exchange sites between spins (*S_i_,S_j_*) and heavy metal site (*l*) result in a non‐zero DMI term (*D_ijl_*) whose sign is dependent on the polarity of the gradient. b) XRD spectra taken on 20 and 30 nm g‐CoPt films with Δ*x* ± 50%. IP and OOP magnetization hysteresis loops of Δ*x* = +50% 20 nm c) and 30 nm d) g‐CoPt films. The corresponding Δ*x* = ‐50% films’ IP and OOP hysteresis loops are shown in e‐f. Effective magnetic anisotropy *K*_eff_ versus thickness for Δ*x* = +50% (inset of d) and Δ*x* = −50% (inset of f) g‐CoPt single layers.

From previous studies,^[^ ^24^, ^25^, ^30^, ^31^ ^]^ there is a proportionality between the effective gradient and the resulting g‐DMI. It is noted that the effective gradient is defined as composition gradient of Co (Δ*x*)/thickness (*t*). Thus, for a fixed value of Δ*x*, the g‐DMI can be modulated by changing *t*. In this study, SiO_2_ (2 nm)/Co*_x_* Pt_1–_ *_x_* (thickness *t* = 10, 20, 30 nm) with fixed gradient parameters of opposite sign (Δ*x* = ±50%) were deposited on a SrTiO_3_ (STO) (111) single‐crystal substrate (see Experimental Section for the growth details).^[^ ^30^ ^]^ Considering the hexagonal close packed (HCP) structure, the lattice constants are 5.19, 5.472, and 5.529 Å for Co_3_Pt, CoPt_3_, and STO substrate respectively. Due to the small lattice mismatch between Co*_x_* Pt_1‐_ *_x_* and STO, STO substrate is selected for the preparation of our g‐CoPt films. The used composition difference Δ*x* of +50% (−50%) corresponds to CoPt_3_ → Co_3_Pt (Co_3_Pt → CoPt_3_) from the start to end of the growth, see **Figure** **1**a. Figure 1b shows X‐ray diffraction (XRD) spectra of the Δ*x* = ±50% 20 and 30 nm thick g‐CoPt films. The XRD spectra of the Δ*x* = ±50% 10 nm g‐CoPt films were reported in our previous work.^[^ ^30^ ^]^ There is significant broadening compared to binary (non‐gradient) CoPt films, which may be affected by nonuniform composition or a lack of long‐range crystallinity.^[^ ^32^, ^33^ ^]^ Atomic force microscopy topography measurements on Δ*x* = ±50% 20 and 30 nm g‐CoPt films revealed smooth surfaces with a roughness in the range of 0.48–1.48 nm (see Section S1.1 and Figure S1.1, Supporting Information).

Energy dispersive X‐ray spectroscopy in a scanning transmission electron microscopy configuration was performed on selected Δ*x*  = +50% g‐CoPt films (thickness of 20 and 30 nm) to confirm the composition gradient with a relative ratio of Co to Pt of 3:1, see Section S1.2 and Figure S1.2 (Supporting Information).

The out‐of‐plane (OOP) and in‐plane (IP) *M*‐*H* hysteresis loops were performed using Superconducting Quantum Interference Device (SQUID) magnetometry and displayed in Figure 1c,f for Δ*x* = +50% (Δ*x* = −50%) 20 and 30 nm g‐CoPt films, respectively. The effective magnetic anisotropy, *K*_eff_ = *M_s_*  × Δ/μ_0_, is determined by taking the difference of area under OOP and IP magnetic hysteresis loops in Figure 1c–f.^[^ ^30^, ^34^ ^]^ *M*_s_ is the saturation magnetization ≈700 kA/m^−1^ for all Δ*x* = ±50% g‐CoPt films. As shown in the insets of Figures 1d,f, *K*_eff_ = 111 ± 1, 134.5 ± 4, and 167 ± 3 kJ/m^3^ for 10, 20, and 30 nm Δ*x* = +50% g‐CoPt films, respectively. *K*_ef_ = 38.4 ± 1.3, 22.3 ± 0.3, and 15.7 ± 0.47 kJ/m^3^ for 10, 20, and 30 nm Δ*x* = −50% g‐CoPt films, respectively. Since the bulk perpendicular magnetic anisotropy (PMA) of CoPt film relies on the close‐packed stackings of Co/Pt, the increase of HCP phase order degree gives rise to the higher *K*_eff_. The stacking order thus influences crystallographic quality due to the lattice mismatch among Co_3_Pt, CoPt_3_, and the STO substrate. If CoPt_3_ layer is initially grown, it could be regarded as the seed layer to promote the subsequent growth of HCP phase of Co_x_Pt_1‐x_ alloy.^[^ ^35^, ^36^, ^37^ ^]^ It results in that *K*_eff_ of Δ*x* = +50% (CoPt_3_→Co_3_Pt) films is higher than Δ*x* = −50% (Co_3_Pt→CoPt_3_) films (see the insets of Figure 1d,f). The relatively lower crystallinity for the Δ*x* = −50% (Co_3_Pt→CoPt_3_) films agrees with the XRD spectra in Figure 1b. Furthermore, increasing the film thickness promotes (suppresses) the HCP phase formation in the Δ*x* = +50% (− 50%) films.

**Figure 2:**
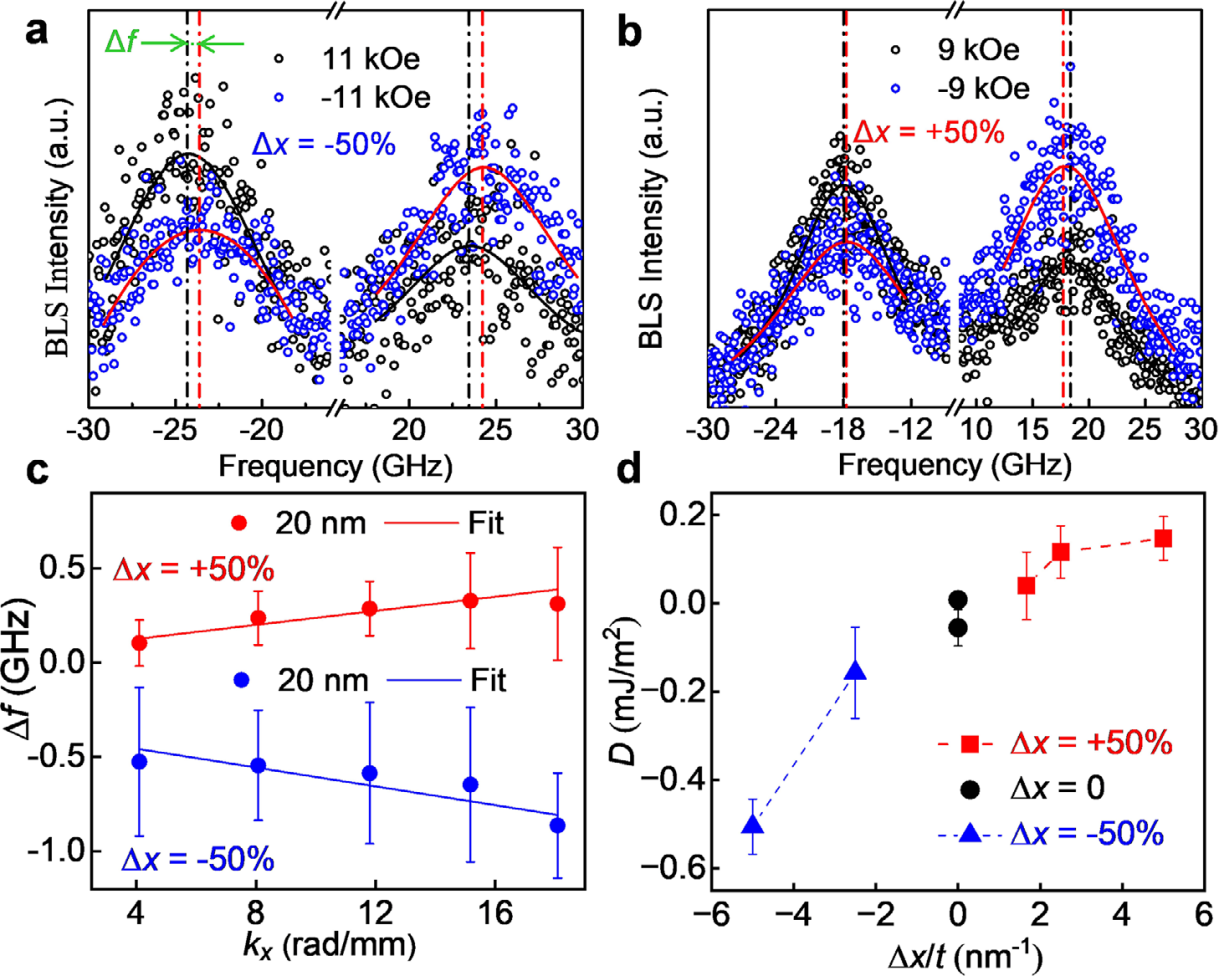
Summary of g‐DMI measurements on g‐CoPt films of different thicknesses: BLS spectra for Δ*x* = −50% a) and Δ*x* = +50% b) 20 nm g‐CoPt film in which the scattered black and blue curves represent the spectra with ± µ_0_ *H* and the solid lines are the fitting curves. c) Δ*f* versus *k_x_* for Δ*x* = ± 50% 20 nm g‐CoPt films. The sign and magnitude of Δ*f*/*k*_x_ is reflected in the calculated g‐DMI energy. d) *D* versus the effective gradient parameter, Δ*x*/*t*, in all graded (10 – 30 nm) and non‐graded CoPt films.

BLS spectroscopy was used to estimate the value of g‐DMI in all Δ*x* = ±50% 10–30 nm g‐CoPt films. The nonreciprocal frequency shift, Δ*f*, between Stokes and anti‐Stokes peaks in the BLS spectra is related to the DMI energy density as:^[^ ^25^, ^30^ ^]^ Δ*f* = 2γ*Dk*_x_/π*M*_s_, where *γ*, *D*, and *k*_x_ are the gyromagnetic ratio, the volume‐averaged DMI constant, and the projection of the spin‐wave vector (***k***) in the direction perpendicular to the applied magnetic field µ_0_ *H*, respectively.^[^ ^30^ ^]^ The representative BLS spectra for negative (positive) gradient 20 nm films are shown in **Figure** **2**a,b. Δ*f* was measured as function of *k*_x_ and plotted in Figure 2c for a selected g‐CoPt films, in which the applied magnetic field is larger than magnetic anisotropy field. Here, the prominent aspects of g‐DMI are in accordance with the previous experimental and theoretical determinations, namely that the sign of Δ*f*, and thus the sign of *D*, is negative for a negative gradient parameter. The magnitude of g‐DMI *D* is plotted versus the effective gradient parameter Δ*x*/*t* in Figure 2d.

As expected, *D* increases with increasing Δ*x*/*t*, from 0.16±0.10 mJ/m^2^ for the negative gradient 20 nm film to 0.51±0.06 mJ/m^2^ for the 10 nm film. For the positive gradient samples, *D* increases from 0.04±0.08 mJ/m^2^ for the 30 nm film to ≈0.15±0.05 mJ/m^2^ for the 10 nm film. The high deviation of DMI values between the positive and negative gradient films may be explained by additional long‐range structural asymmetries along the thickness direction in the film, such as crystal phase, and magnetic anisotropy, which compromises the coherence and lifetime of spin waves and consequently reduces the BLS signal strength while increasing the noise. For the 30 nm films, a measurable value of Δ*f* could only be obtained from Δ*x* = +50% g‐CoPt (see Section S2, Supporting Information), showing a magnitude of *D* of 0.04±0.08 mJ/m^2^, almost comparable with ungraded films (D = 0.055±0.041 mJ/m^2^ for Co_3_Pt and = 0.008±0.001 mJ/m^2^ for CoPt_3_).^[^ ^30^ ^]^ Bloch‐type skyrmions can be stabilized in the 10 nm g‐film films by g‐DMI,^[^ ^30^ ^]^ however for thicker g‐CoPt films (e.g., Δ*x* = +50% 30 nm), skyrmions can be still stabilized even with close to zero g‐DMI. We note the absence of magnetic skyrmions in the non‐graded CoPt_3_ and Co_3_Pt, suggesting that g‐DMI is needed to stabilize them.^[^ ^30^ ^]^

### 2.2 The Evolution of Spin Textures via MFM Imaging

**Figure 3:**
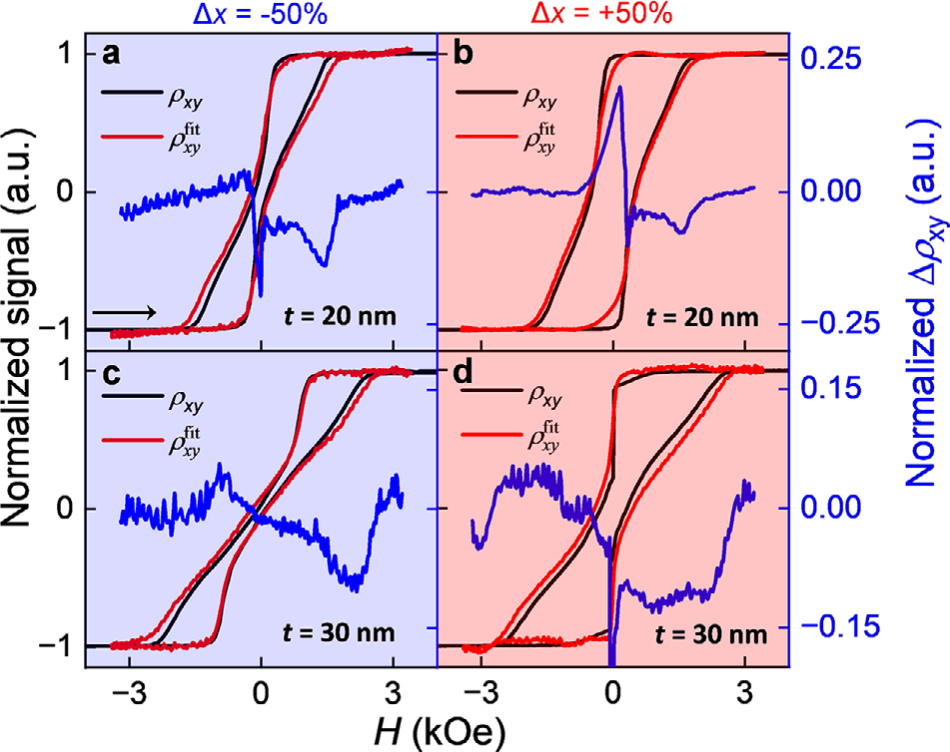
Extraction of the residual resistivity Δρ_*xy*_ from Hall effect and MOKE measurements. In each plot, Δρ_*xy*_(*H*) (blue line) includes the THE signal which is obtained by subtracting ρ_*xy*_(*H*) measured by PPMS (red line) from $\rho_{xy}^{fit}\left(H\right)$ fitted from MOKE measurements (black line) for one direction of the applied magnetic field sweep (indicated by arrow in a). a,b) Extracted Δρ_*xy*_(*H*) signal for 20 nm g‐CoPt films with a composition gradient of Δ*x* = −50% and Δ*x* = +50%, respectively. c,d) Extracted Δρ_*xy*_(*H*) for 30 nm g‐CoPt films of Δ*x* = −50% and Δ*x* = +50%, respectively.

We next investigated the evolution of topological spin textures by independent magneto transport (Physical Property Measurement System, PPMS) and magneto‐optical Kerr effect (MOKE) measurements. The interaction between skyrmions and charge carriers gives rise to the topological Hall effect (THE), which is reflected in the residual resistivity Δρ_*xy*_ (*H*) =  ρ_*xy*_(*H*) − [*R*_0_ *H* + *R_s_M*(*H*)], where ρ_*xy*_(*H*) is the total Hall resistivity, and *R*_0_ *H* and *R_s_M*(*H*) are the ordinary and anomalous Hall contributions, respectively. The residual resistivity Δρ_*xy*_(*H*) was estimated by subtracting the fitted background $\rho_{\mathrm{xy}}^{\mathrm{fit}}\;\left(H\right)$ from the measured ρ_*xy*_(*H*). Here, ρ_*xy*_(*H*) was obtained from PPMS transport measurements, while *M*(*H*) was derived from MOKE measurements (see Experimental Section and References^[^ ^30^, ^38^ ^]^ for further details). Since the transport and MOKE measurements were performed using different setups, we compensated for the mismatch in magnetic field calibration using a scaling factor of 0.85. See Section 3 and Figure S3.1 (Supporting Information) for further details. The normalized ρ_*xy*_(*H*), $\rho_{xy}^{fit}\left(H\right)$, and Δρ_*xy*_(*H*) for 20 and 30 nm g‐CoPt films are plotted in Figure 3, with the constituent measurements plotted for reference. It is worth noting that the plotted curves only represent one magnetic field sweep direction, which is denoted by the arrow in **Figure** **3**a. From the plots of Δρ_*xy*_(*H*), there appears to be non‐zero signal for each film. These signals may not arise solely from THE. Contributions from polydomain states and shape anisotropy introduced by the Hall bar geometry may also be involved. Notably, there is an asymmetry in the magnitude of Δρ_*xy*_(*H*) peaks forming from uniform magnetization (decreasing magnetic field) as compared to from the polydomain state (increasing magnetic field).^[^ ^30^ ^]^ The evolution of topological spin textures inferred from the Δρ_*xy*_(*H*) signal is further supported by MFM imaging.^[^ ^38^ ^]^

**Figure 4:**
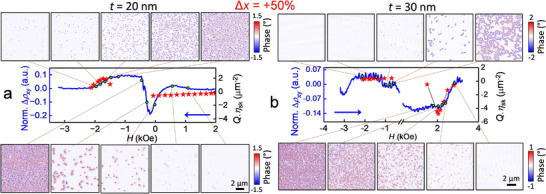
Correlation of MFM imaging and Hall measurement residual. a) Comparison of MFM and THE measurements on Δ*x* = +50% 20 nm g‐CoPt film. The direction of the magnetic field sweeps for both imaging and extracted THE are indicated by the blue arrow. The estimated topological charge density, *Q* · *n_Sk_* is plotted as red stars. The green dots show the magnetic field at which the corresponding MFM images were taken. b) Comparison of MFM and THE measurements on 30 nm, Δ*x* = +50% g‐CoPt film. Note that the direction of field sweep is reversed from (a).

To obtain a clearer understanding of the underlying magnetization patterns which can give rise to THE signals, we performed MFM imaging of the magnetization reversal process over the same field sweep conditions as were used to obtain Δρ_*xy*_(*H*) curves. The results for Δ*x* = +50% g‐CoPt films (*t* = 20, 30 nm) are shown in **Figure** **4**. Again, it is worth noting the direction of the magnetic field sweep, which is shown in the Δρ_*xy*_(*H*) plots as the blue arrows. Upon reduction of the amplitude of applied magnetic field *H* in the uniform state, small isolated spin textures emerge which may or may not possess a topological charge. From the MFM images, it is not possible to characterize the topology of such bubbles, and instead the density is estimated by indiscriminately counting isolated spin textures. Applying the product of relative topological charge, *Q*, the topological charge density *Q*⋅*n_Sk_* can be roughly compared with the Δρ_*xy*_(*H*) curves. Taking Figure 4a for 20 nm g‐CoPt film, the amplitude and horizontal axis position of the roughly estimated topological density, there is some overlap with the Δρ_*xy*_(*H*) curves, with the asymmetric behavior depending on the initial condition being captured as well. The asymmetry is even more pronounced in the 30 nm g‐CoPt film case, where the density of skyrmions nucleated from the polydomain phase is around one order of magnitude higher than that of those nucleating from the uniform state. The comparison of MFM and Δρ_*xy*_(*H*) curves is also shown for the Δ*x* = −50% 20, 30 nm g‐CoPt films in Section S3 and Figure S3.2 (Supporting Information). Interestingly, the 30 nm Δ*x* = −50% g‐CoPt film shows a distinctly low density of isolated skyrmions, though still has a prominent THE‐like peak, which may stem from alternative mechanisms for topological Hall‐like signals including chiral domain walls, Berry curvature, multiple hysteresis loops, or pinning effects.^[^ ^39^ ^]^ Despite the qualitative agreement between THE and skyrmion density in positive graded films, there is not a direct prediction of the skyrmions density according to the emergent field model in the strong coupling regime, where the extracted THE resistivity component can be directly related to the density of topological spin objects.^[^ ^40^ ^]^ This is likely due to the role of spin polarized carrier density in magneto‐transport measurements,^[^ ^41^ ^]^ as well as the residual resistivity Δρ_*xy*_ not solely related to THE (includes contributions from pinning effects induced by the edges of the Hall bar pattern) which render the simplified model insufficient. The lack of isolated skyrmions was also observed on Δ*x* = −50% 30 nm g‐CoPt film grown on a Al_2_O_3_ substrate (see Figure S3.3, Supporting Information). It is likely that the formation of skyrmions in these films is energetically unfavorable due to the lack of PMA. In this case, the energy barrier is likely too small to support skyrmions at room temperature and the system will favor the formation of the labyrinth domains.^[^ ^42^ ^]^

### 2.3 Micromagnetic Simulations and Energy Barrier Calculations

We used micromagnetic simulations to gain an additional insight of the role of film thickness on the stability, helicity, and size of spin textures in g‐CoPt films using the GPU‐based platform Mumax3.^[^ ^43^ ^]^ In the simulation the difference between positive and negative gradient cases comes from the difference in magnetic anisotropy. The uniaxial anisotropy density (*K*_u_) used in the simulations is related by *K_u_ = K_eff_ + µ_0_M_s_^2^*/2 to the effective magnetic anisotropy *K_eff_* measured in experiments (discussed above), and magnetic dipolar interactions were switched on in simulations. In the micromagnetic simulations we used the following parameters: Exchange stiffness *A*_ex_ = 10 J/m and mesh size is taken to be 2.5 nm × 2.5 nm × 2.5 nm. The simulations are done at zero temperature. For the minimum energy path (MEP) calculations,^[^ ^44^ ^]^ Gilbert damping α = 1 is used to control the energy minimization at each step.

**Figure 5:**
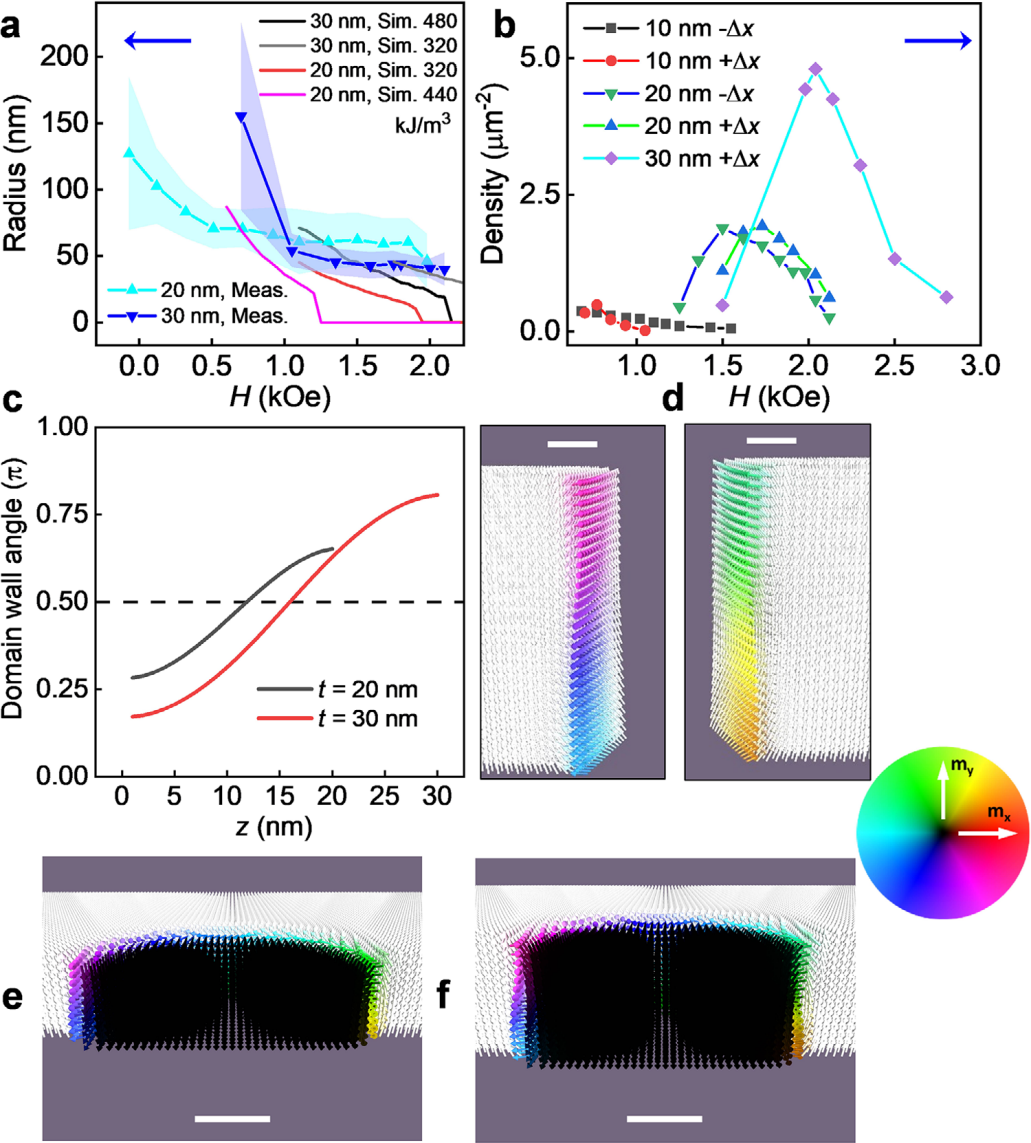
Summary of MFM and micromagnetic simulations of skyrmions in g‐DMI films. a) Measured (blue and aqua lines) and simulated (purple and black lines) skyrmion radius versus *H* for Δ*x* = +50% g‐CoPt of 20, 30 nm thickness. Red and grey lines are with *K*_u_ at the shape demagnetization limit for 20 nm and 30 nm thickness (*K*_u_ = 320 kJ m^3^). The experimental data is shown with a shaded area which represents the interquartile range of the measured radii. b) Density of skyrmions from MFM imaging for 10, 20, 30 nm thick g‐CoPt. Blue arrows in (a) and (b) indicate the magnetic field sweep direction. c) Simulated twisting of the domain wall angle through the thickness for Δ*x* = +50% g‐CoPt of 20 nm thickness with *K*_u_ = 440 kJ m^3^, DMI = 0.12 mJ m^2^ and 30 nm thickness with *K*_u_ = 480 kJ m^3^, g‐DMI = 0.05 mJ m^2^. d) Vector representation of cross‐section of the helical skyrmion domain walls. Cross‐section view of skyrmion for 20 nm e) and 30 nm f) Δ*x* = +50% g‐CoPt films. The white arrows are the uniform background, and the black arrows are the core of the skyrmion. The scale bar in (d) is 5 nm and in (e‐f) is 10 nm.

From magnetic simulations, we find that the main effect of increasing the thickness of the film, especially in the case of Δ*x* = +50% g‐CoPt films, is to increase the relative contribution of magnetic dipole‐dipole interactions. In addition, g‐CoPt films with different thicknesses exhibited variations in magnetic anisotropy (discussed in Section 2.1). Both of the above have implications for the magnetic field range in which skyrmions stabilize, as well as for their energy barrier to decay into either the uniform or maze domain phase. To account for the effect of magnetic anisotropy, we performed micromagnetic simulations for a range of values in the vicinity of the measured magnetic anisotropy values. In **Figure** **5**a, the estimated skyrmions radius from MFM images (Figure 4; Figure S3.2, Supporting Information) is compared with the radius of skyrmions from simulations as a function of external magnetic field for Δ*x* = +50%g‐CoPt films. The simulations qualitatively agree with the experimental findings, exhibiting a threshold field above which skyrmions collapse, as evidenced in Figure 5b.

Furthermore, the micromagnetic simulations presented in the Section S4 (Supporting Information) show that at lower magnetic fields, the energetically preferred maze state takes over, which is consistent with Figure 5b, showing the presence of skyrmions within a specific range of magnetic fields,^[^ ^45^, ^46^ ^]^ see for example Figure S4.3 (Supporting Information). We observe some discrepancy between the experimentally determined range of magnetic fields in which skyrmions are stable and the micromagnetic simulations, as evidenced in Figure 5b, particularly for the Δ*x* = +50% 20 nm g‐CoPt film. These discrepancies are likely related to nonuniformities in the graded film, as well as to the presence of pinning centers, which create higher energy barriers to annihilation^[^ ^47^ ^]^ which can affect the stability of magnetic skyrmions. Other differences may be accounted for by the fact that simulations were performed without the influence of temperature.^[^ ^6^ ^]^ Further details on the effect of g‐DMI amplitude on the radius and helicity of skyrmions, as well as plots of skyrmion radius versus *H* for different anisotropy values, are provided in Section S4 and Figure S4.1–S4.3 (Supporting Information). The estimated density of skyrmions when sweeping from the labyrinth phase to saturation in g‐CoPt films of 10, 20, 30 nm thicknesses are shown in Figure 5b, deduced from MFM images in Figure 4, Figure S3.2 (Supporting Information), and reference^[^ ^30^ ^]^ (for 10 nm films). For Δ*x* = +50% g‐CoPt films the combined effect of increased dipolar interactions and magnetic anisotropy results in higher skyrmion densities, as previously discussed in the context of the comparison to THE measurements. It is known that magnetic dipolar interactions can favor the formation of Bloch‐type skyrmions.^[^ ^42^ ^]^ Since other magnetic textures exist in the experiment as well, individual skyrmions can be affected by other magnetic textures which can further increase their stability, as evidenced in simulations.^[^ ^30^ ^]^ Unlike g‐DMI, the lack of a well‐defined handedness of dipolar interactions can give rise to a wider variety of spin textures including higher order skyrmions and antiskyrmions.^[^ ^30^ ^]^

In the Section S5 (Supporting Information), we analyze the likelihood of the appearance of other textures by calculating the MEP using the string method,^[^ ^44^ ^]^ see for example Figure S5.1 (Supporting Information), demonstrating substantial preference for the formation of skyrmions over trivial bubbles. We therefore expect that most of the observed textures in our MFM images are skyrmions as evidenced from Figure S4.4 (Supporting Information). Importantly, the densities shown are those that emerge when the magnetic field is increased from the polydomain state. The micromagnetic simulations in Figure S4.2–S4.4 (Supporting Information), as well as the MEP calculations in Figure S5.2 (Supporting Information), suggest that the energy cost for skyrmion formation from the polydomain state differs from that of the transition from the uniform magnetization state. In particular, when we start from the maze state, the energy cost for forming a skyrmion is close to zero at a certain magnetic field. When we start from the uniform state, there is always a finite energy cost associated with skyrmion formation. These differences in energetics may be correlated with the resulting skyrmion density, i.e., overcoming larger energy barrier may be less probable. This correlates well with the MFM results in Figure 4, where a higher density of skyrmions is observed when sweeping from the polydomain state.

For thicker simulated g‐CoPt single layer, an increasingly significant rotation of the domain wall angle from the bottom to the top interface was also observed in micromagnetic simulations. This is shown in Figure 5c, where the domain wall angle is plotted versus thickness in units of π. The dashed line indicated where the helicity is Bloch‐type, and the plot bounds correspond to the Néel‐type. Thus, thicker films approach Néel‐type helicity at the interfaces due to the minimization of stray field energy. The simulated vectorial representations of cross sections of skyrmion domain wall magnetization are shown in Figure 5d for 30 nm Δ*x* = +50%. Such skyrmions with twists have been observed experimentally.^[^ ^48^ ^]^ Figure 5e,f shows the cross section of skyrmion for 20 nm (30 nm) Δ*x* = +50% g‐CoPt film and illustrates the helicity twisting. The twisted helicity in thicker films could have implications for skyrmion mobility in response to applied current density.

## 3 Conclusion

In closing, we studied a series of g‐CoPt single layers of different thicknesses (10 – 30 nm) and characterized the stability of room temperature skyrmions. First, we find that the Δ*x* = +50% g‐CoPt films possess THE residuals which correlate with the density of skyrmions observed from MFM imaging, though there is a discrepancy between these measurements for the case of Δ*x* = +50% 30 nm g‐CoPt film. The asymmetry of skyrmion nucleation density is also reflected in the measurements as a function of the magnetic field‐sweep direction, which may be related to the difference in energy barrier between the skyrmion phase and the starting spin configuration. We also observed that the magnetic dipolar interactions in thick g‐CoPt films can drive the formation of a higher density of skyrmions even though g‐DMI is reduced with increasing the film thickness.

The imperfect alignment of the topological Hall effect contribution and the measured skyrmion density is attributed to sample inhomogeneity and additional topological Hall signatures including domain walls. These results aim to provide clarity regarding the stability of topological spin textures in samples with g‐DMI and may help to inform the appropriate applications of skyrmions in compositional gradient films and directions for subsequent experiments. Examples of such follow up experiments include monitoring the skyrmion Hall angle upon injection of current^[^ ^49^ ^]^ as well as investigation of the extreme cases of effective gradient, where g‐DMI is either maximized or negligible and can be used to control the helicity of skyrmions.^[^ ^25^, ^30^, ^50^ ^]^

## 4 Experimental Section

Compositional gradient engineered CoPt, g‐CoPt, (thickness *t* = 10, 20, and 30 nm) films were grown on STO (111) substrates by using d.c. and radio‐frequency magnetron sputtering (Kurt J. Lesker). The relative deposition rates of the Co and Pt elements were linearly changed during the growth, leading to a linear composition‐magnetization change along the growth direction.^[^ ^30^ ^]^ During the growth, the temperature and the Ar gas pressure were kept constant at 280 °C and 6 mTorr, respectively. A 2‐nm SiO_2_ capping layer was then deposited by radio‐frequency magnetron sputtering to prevent any oxidation effect after the g‐CoPt films were cooled down to room temperature. Magnetic properties were measured by a SQUID (Quantum design MPMS3). X‐ray diffraction measurements were performed at room temperature at the Singapore Synchrotron Light Source with an X‐ray wavelength of 1.541 Å. The CoPt films were patterned into a Hall bar with a width of 10 µm by using an Ultraviolet Maskless Lithography machine (TuoTuo Technology) and the ion beam etching technology.

THE signal ρ_*TH*_(*H*) was extracted based on the relation: ρ_*xy*_ (*H*) =  *R*_0_ *H* + *R_s_M*(*H*) + ρ_*TH*_(*H*), where *R*_0_ *H* and *R_s_M*(*H*) are the ordinary and anomalous Hall components, respectively, and ρ_*TH*_(*H*) is the resistivity from the contribution of THE. ρ_*xy*_(*H*) was measured using PPMS with a DC current of 200 µA, under a swept out‐of‐plane magnetic field *H*. The residual resistivity Δρ_*xy*_(*H*) was estimated by subtracting the fitted background $\rho_{xy}^{fit}\;\left(H\right)=R_{0}H+R_{s}M\left(H\right)$ from the experimental data, which included the contribution of THE signal ρ_*TH*_(*H*). The magnetic hysteresis loops *M*(*H*) for g‐CoPt single layers were measured using MOKE at the center of the Hall bar.

## Conflict of Interest

The authors declare no conflict of interest.

## Author Contributions

A.E., Q.Z., H.V. and E.S. contributed equally to this work. A.E. performed MFM measurements and analyzed the data with assistance of S.L. and S.‐H.L.; Q.Z, grew the g‐CoPt films and performed topography, XRD, MOKE, SQUID, anomalous Hall effect, and THE measurements; H.V., E.S., and A.K. performed micromagnetic modeling and skyrmion relaxation/energy barrier calculations; C.L. and G.C. performed BLS measurements; B.L. and D.S. performed high‐angle annular dark‐field scanning transmission electron microscopy and energy dispersive X‐ray spectroscopy on the g‐CoPt films; A.L., J.C., and A.K. designed the experiments and supervised the project. A.E. and A.L. wrote the paper with contributions and feedback from all authors.

## Supporting information



Supporting Information

## Data Availability

The data that support the findings of this study are available from the corresponding author upon reasonable request.
