# Supplementary material for: Effect of Magnetic Anisotropy and Gradient‐Induced Dzyaloshinskii‐Moriya Interaction on the Formation of Magnetic Skyrmions
Source: Small. 2025 Jul 26;21(37):e05204. doi: 10.1002/smll.202505204 (PMC12444826; doi:10.1002/smll.202505204)
Supplement: Supplementary file 1 — Supporting Information [file SMLL-21-e05204-s001.pdf]

NANO · MICRO
small

Supporting Information

for *Small*, DOI 10.1002/smll.202505204

Effect of Magnetic Anisotropy and Gradient-Induced Dzyaloshinskii-Moriya Interaction on the Formation of Magnetic Skyrmions

Adam Erickson, Qihan Zhang, Hamed Vakili, Edward Schwartz, Suvechhya Lamichhane, Chaozhong Li, Boyu Li, Dongsheng Song, Guozhi Chai, Sy-Hwang Liou, Alexey A. Kovalev, Jingsheng Chen* and Abdelghani Laraoui**

Supporting Information

Effect of Magnetic Anisotropy and Gradient-Induced Dzyaloshinskii-Moriya Interaction on the Formation of Magnetic Skyrmions

Adam Erickson,[1†] Qihan Zhang,[2†] Hamed Vakili,[3†] Edward Schwartz,[3†] Suvechhya Lamichhane,[3] Chaozhong Li,[4] Boyu Li,[5] Dongsheng Song,[5] Guozhi Chai,[4] Sy-Hwang Liou,[3] Alexey A. Kovalev,[3] Jingsheng Chen,[2,6*] Abdelghani Laraoui[1,3*]*

[1]Department of Mechanical & Materials Engineering, University of Nebraska-Lincoln, 900 N 16th Street, W342 NH, Lincoln, NE 68588, United States
[2]Department of Materials Science and Engineering, National University of Singapore, Block E2, #05-19, 5 Engineering Drive 2, Singapore 117579, Singapore
[3]Department of Physics and Astronomy and the Nebraska Center for Materials and Nanoscience, University of Nebraska-Lincoln, 855 N 16th St, Lincoln, NE 68588, United States
[4]Key Laboratory for Magnetism and Magnetic Materials of Ministry of Education, School of Physical Science and Technology, Lanzhou University, Lanzhou 730000, China
[5]Institutes of Physical Science and Information Technology, Anhui University, Hefei, 230601, China
[6]National University of Singapore (Suzhou) Research Institute, Suzhou, Jiangsu, 215123, China
[†]Equal contributions
[*]Corresponding authors: akovalev2@nebraska.edu, msecj@nus.edu.sg, alaraoui2@unl.edu

S1.1 Topography of gradient-composition engineered CoPt (g-CoPt) films

Atomic force microscope (AFM) images acquired from $\Delta x = \pm 50\%$ 20 and 30 nm thick g-CoPt films grown on $SrTiO_3$ (STO) substrate are shown in Figure S1.1 with the root mean square surface roughness, R_q, of each image, indicated in the inset. While the 20 nm, $\Delta x = +50\%$ film showed a larger (~1.48 nm) surface roughness, the magnetic textures did not exhibit any correlation to topography. The other films displayed sub-nanometer roughness, less smoother than $\Delta x = \pm 50\%$ 10 nm g-CoPt films (Ra ~110 – 234 pm) studied in reference [1].

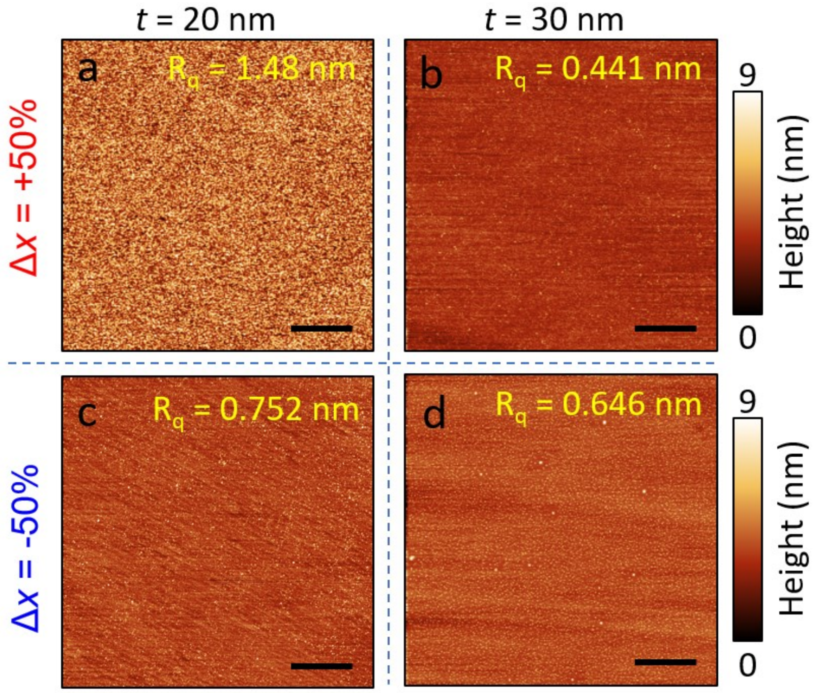

Figure S1.1: AFM images of 20 nm (a) and 30 nm (b) $\Delta x = +50\%$ g-CoPt films. AFM images of 20 nm (c) and 30 nm (d) $\Delta x = -50\%$ g-CoPt. The scale bar in all images is 2 μm.

S1.2 Structural characterization of g-CoPt films

To map the composition gradient of the g-CoPt films, we performed energy dispersive X-ray spectroscopy (EDS) in a scanning transmission electron microscopy (STEM) configuration on selected $\Delta x = +50\%$ g-CoPt films (thickness of 20 nm and 30 nm). The sample specimens were prepared by Ga focused ion beam (FIB) milling. The EDS spectra are obtained on a probe/image aberration-corrected scanning transmission electron microscope (STEM). The EDS maps shown in Figure S1.2a for 20 nm film and Figure S1.2c for 30 nm film confirm the presence of Co and Pt gradient through the film thickness. The normalized EDS intensity, integrated across the areas in Figure S1.2b (for the 20 nm film) and Figure S1.2b (for the 30 nm film), show the evolution of the composition of Co and Pt through the thickness of the g-CoPt films with a relative ratio of Co to Pt is approximately 3:1, which agrees with theoretical values and with those measured in $\Delta x = \pm 50\%$ 10 nm g-CoPt films.[1] The compositional gradient leads to bulk magnetic asymmetry and gradient Dzyaloshinskii–Moriya interaction (g-DMI). [1–3]

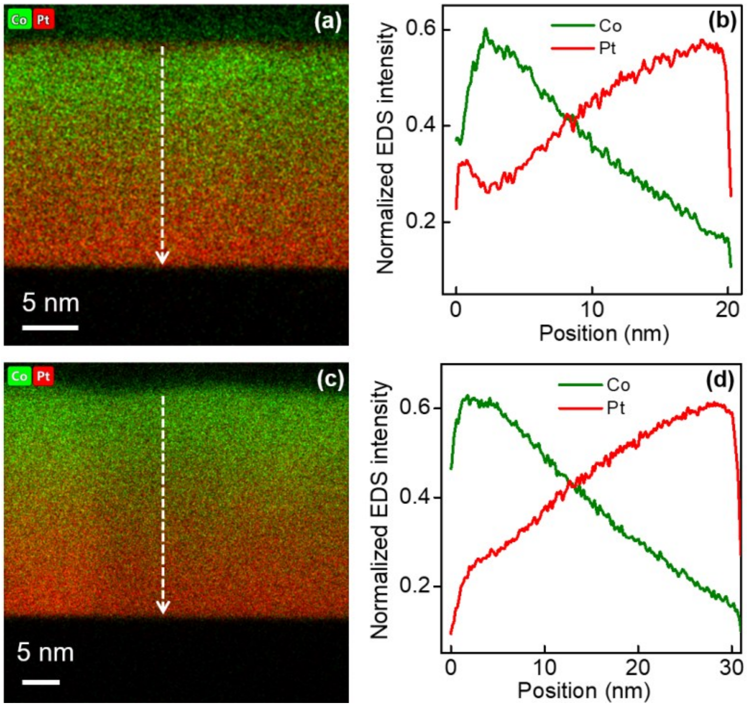

Figure S1.2: STEM-EDS image of Δx = +50% 20 nm (a) and 30 nm (c) thick g-CoPt films with plotted contribution of Co (green) and Pt (red). Normalized EDS intensity for Pt (red solid line) and Co (green solid line) for Δx = +50% 20 nm (b) and 30 nm (d) thick g-CoPt films, measured over the entire area along the arrows in (a) and (c), respectively.

S2. Representative Brillouin light scattering (BLS) measurements of 30 nm g-CoPt films

Due to the relationship between net g-DMI and effective gradient $\Delta x/t$, the non-reciprocal frequency shift Δf between the stokes and anti-stokes peak becomes almost indistinguishable in the measurement for Δx = +50% 30 nm g-CoPt film (see Figure S2.1a). For Δx = -50% 30 nm g-CoPt film (Figure S2.1b), the BLS intensity is very weak to extract Δf, that may be explained by the multidomain state reduces the ordered propagation of magnons.

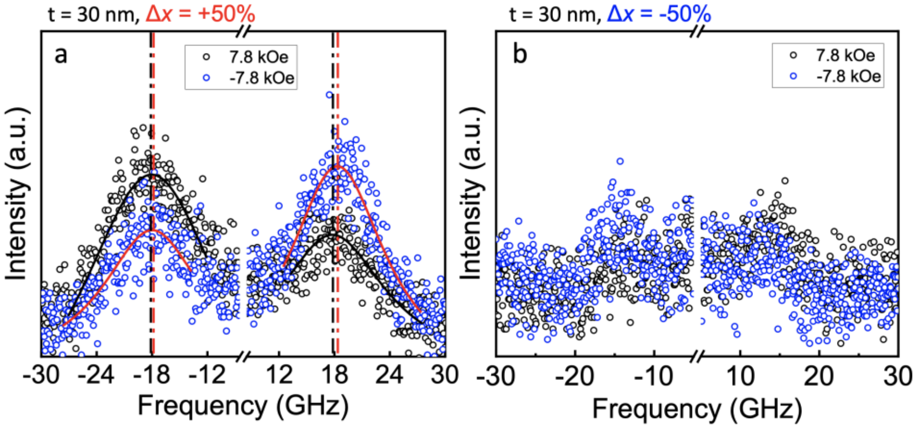

Figure S2.1: BLS spectra of 30 nm, Δx = +50% (a) and Δx = -50% (b) g-CoPt films. Due to the low signal to noise ratio, an estimation of DMI could not be obtained for Δx = -50% g-CoPt.

S3. MFM imaging and $\Delta\rho_{xy}(H)$ curves in g-CoPt thin films with Δ*x* = -50%

To measure $\Delta\rho_{xy}(H)$ correctly, we calibrated both Physical Property Measurement System (PPMS) and magneto-optical Kerr effect (MOKE) setups using a reference FePt single layer with a strong perpendicular magnetic anisotropy (PMA). The coercive fields of FePt should be identical when measured in both systems, thus enabling us to calibrate the magnetic field scales accordingly. Figures S3.1a and S3.1b present the repeatedly measured MOKE hysteresis loops and Hall resistance curves at different regions of the sample, respectively, with sweep rates of 25 Oe/s for MOKE and 20 Oe/s for Hall measurements. We also varied the field sweep direction during the measurements. To account for differences in field strength between the PPMS (used for transport measurement) and the MOKE system, we compared the coercive fields extracted from the averaged MOKE and Hall curves. A consistent scaling relation was observed: $H_{\mathrm{MOKE}} \times 0.85 = H_{\mathrm{PPMS}}$. Based on this, we applied a field correction factor to analyze the residual Hall resistivity $\Delta\rho_{xy}$.

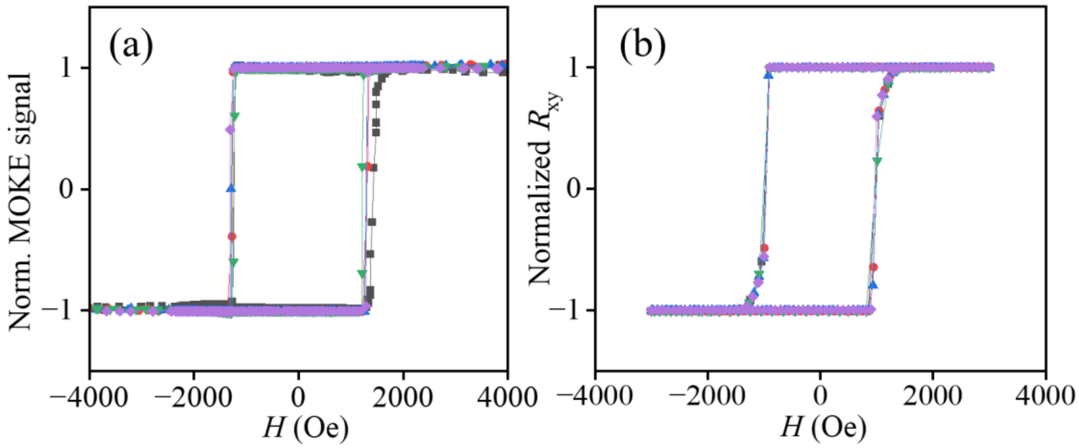

Figure S3.1: (a) MOKE hysteresis loops and (b) Hall resistance curves taken at different regions of the FePt film (scattered curves).

Characterization by $\Delta\rho_{xy}(H)$ curves included topological hall effect (THE) and magnetic force microscopy (MFM) measurements were also conducted on Δ*x* = -50% 20 nm (Figure S3.2a) and 30 nm (Figure S3.2b) g-CoPt/STO films. The size and density of the spin textures are discussed in the main text. The density of skyrmions in Δ*x* = -50% 30 nm g-CoPt film is very low (~0.4 μm^{-2}) in comparison to +50% 30 nm g-CoPt (~5 μm^{-2}). This correlates well with BLS measurements, where g-DMI cannot be extracted in Δ*x* = -50% films due to the weak magnetic anisotropy (discussed above). However, the $\Delta\rho_{xy}(H)$ amplitude of both Δ*x* = ±50% films is comparable, confirming the challenges of using only $\Delta\rho_{xy}(H)$ curves to extract information about chiral spin textures.[4]

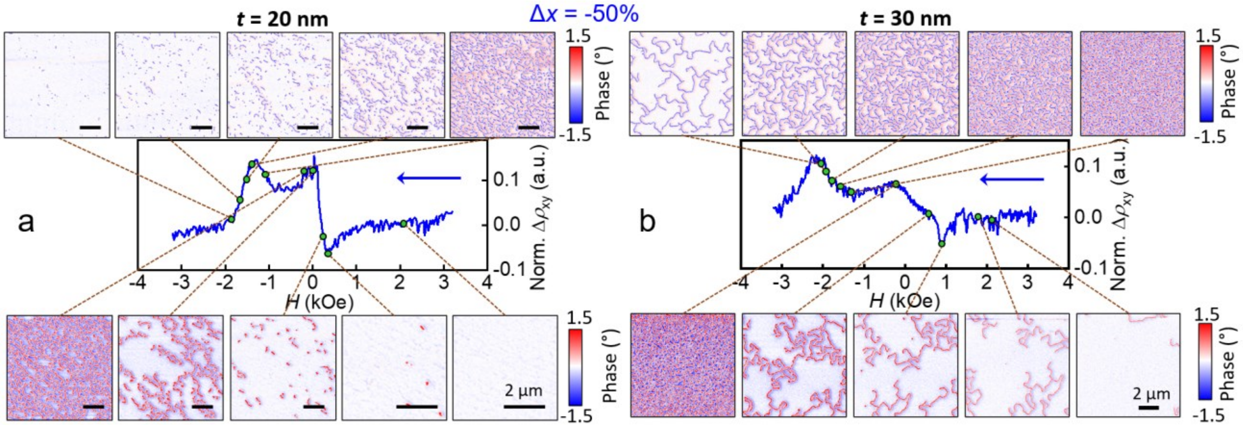

Figure S.3.2: Correlative THE and MFM of 20 nm (a) and 30 nm (b) Δx = -50% g-CoPt films. Blue arrows represent the field sweep directions of both THE and MFM. The scale bars in all MFM maps in (a) is 2 µm.

MFM measurements conducted on Δx = -50% 30 nm g-CoPt film grown on sapphire (Figure S3.3) confirm the absence of magnetic skyrmions that may be explained by the effect of substrate (lattice mismatch) in particular for the $Co_3Pt \rightarrow CoPt_3$.[1]

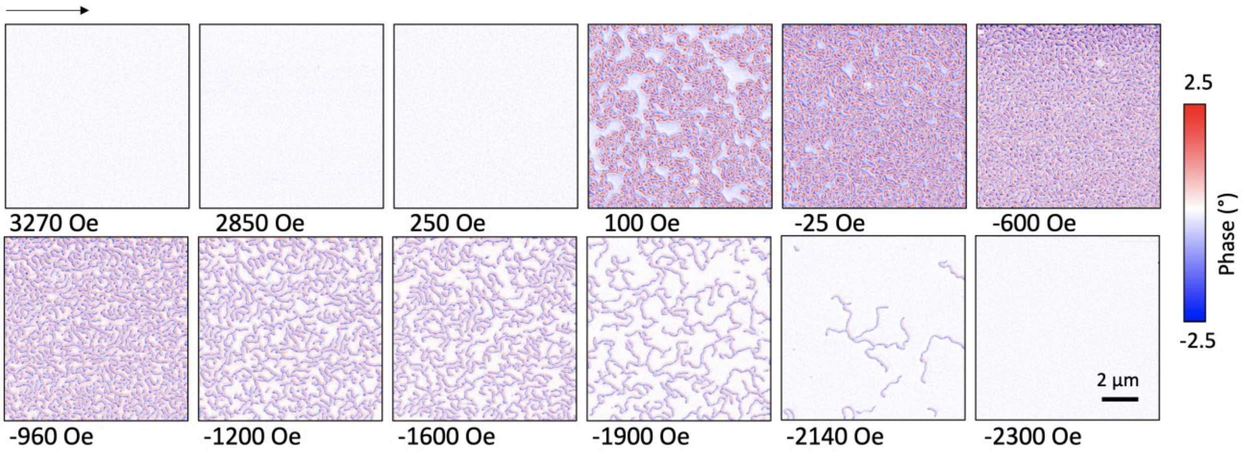

Figure S3.3: MFM images of 30 nm, Δx = -50% g-CoPt grown on Al_2O_3 substrate. The black arrow represents the field sweep direction of MFM measurements

S4. Additional Micromagnetic Simulations

To investigate the stability of skyrmions under various applied magnetic fields and g-DMI strengths, we simulated phase diagrams showing the skyrmion radius and average domain wall (DW) angle, as presented in Figure S4.1, where isolated skyrmions in our simulations could be well represented as 360 degrees DWs. As expected from skyrmion energetics, an applied magnetic field (parallel to the ferromagnetic background magnetization) decreases the skyrmion radius. Above a certain field strength, the skyrmion state collapses (i.e., the radius vanishes), as observed experimentally from nitrogen vacancy (NV) measurements in 10 nm g-CoPt films.[1] Conversely, if the applied magnetic field is too low, maze states are energetically preferred over isolated skyrmions (indicated by the dark red regions in Figures S4.1a and S4.1c).

Figures S4.1b and S4.1d illustrate that increasing the g-DMI strength decreases the average DW angle, bringing it closer to the Néel type. This indicates a transition region from Bloch-type towards Néel-type character, corresponding to hybrid skyrmions.[5] Furthermore, as the applied magnetic field H increases, the skyrmion shrinks, thereby reducing dipolar energy contribution relative to the DMI energy. This effect also contributes to a decrease in the DW angle (a shift towards Néel type). This behavior arises because the dipolar energy contribution favors Bloch-type DWs, while the DMI contribution favors Néel-type DWs.[6] The competition between these energy terms determines the equilibrium DW angle, often resulting in a hybrid skyrmion state in our simulations.

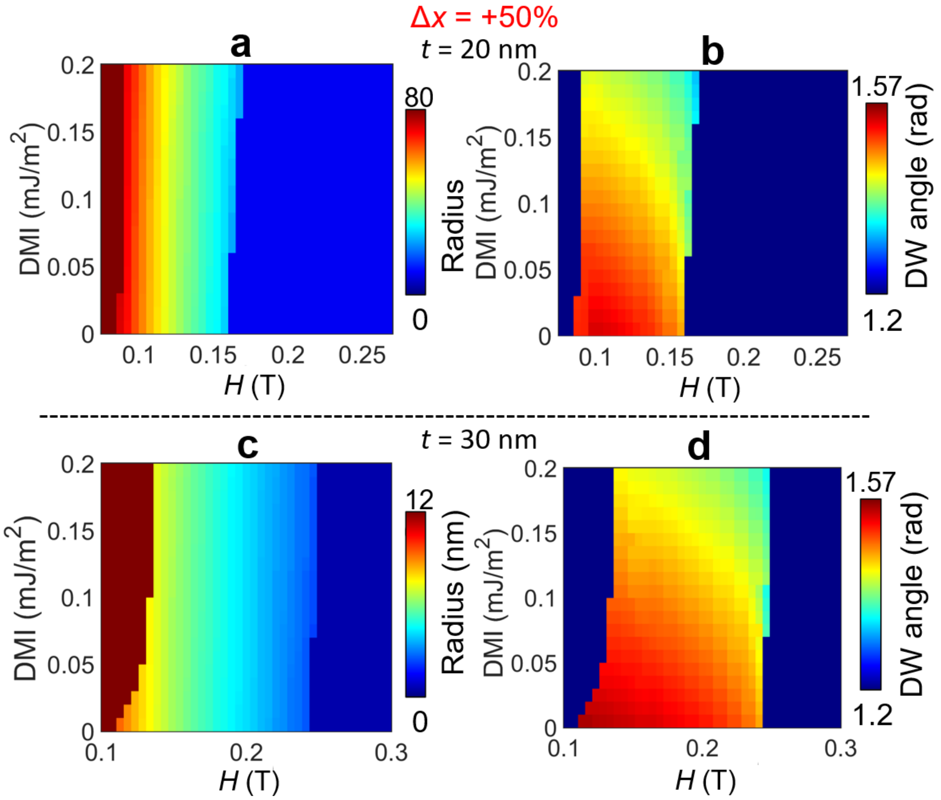

Figure S4.1: Systematically simulated DMI vs H phase diagrams. Calculated radius (a) and domain wall angle (b) of Δx = +50% 20 nm g-CoPt film (K_u =440 kJ/m^3) as a function of g-DMI strength and applied magnetic field amplitude. Calculated radius (c) and domain wall angle (d) of Δx = +50% 30 nm g-CoPt films (K_u =480 kJ/m^3) as a function of g-DMI strength and applied magnetic field amplitude.

In Figure S4.2, we examine the dependence of the skyrmion radius on the applied magnetic field H for different effective magnetic anisotropy density (K_u) values. Increasing K_u lowers the minimum applied magnetic field required to stabilize the skyrmion state relative to the maze state. Conversely, the collapse field (the field above which the skyrmion is no longer stable, i.e., the radius approaches zero) increases as K_u decreases. The simulation dimensions are 1.2 µm × 1.2 µm × 20 nm (30 nm).

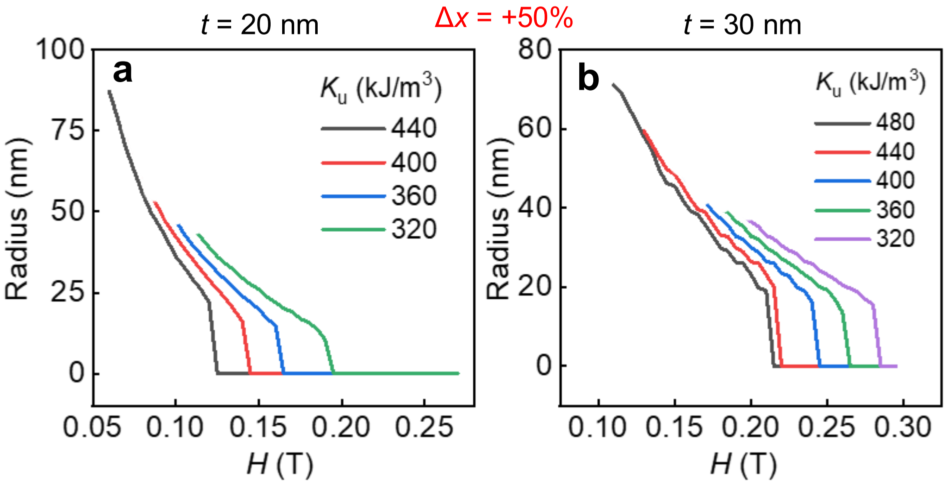

Figure S4.2. Skyrmion radius vs applied magnetic field for varying effective anisotropy in 20 nm (a) and 30 nm (b) Δx = +50% g-CoPt 20 nm correspond to K_u =440 kJ/m^3 and Δx = +50% g-CoPt 30 nm correspond to K_u = 480 kJ/m^3.

Next, we use micromagnetic simulations to assess the range of magnetic fields in which isolated skyrmions can be observed, establishing the low-field bound. Figure S4.3 shows the area density of the energy difference between maze state and uniform state over the simulation system size (1.2 μm × 1.2 μm). The negative values of the energy difference at small magnetic fields indicate that the maze state is more stable than the uniform state. As a result, in this range of magnetic fields, one cannot consider isolated skyrmions in the traditional sense, as they typically arise as metastable states on top of the uniform state. Nevertheless, in this region of magnetic fields one might expect coexistence of skyrmions and maze domains. This behavior agrees with some of our experimental findings where for some applied fields both skyrmion and maze domains coexist in the system, see, e.g., Figure S3.1.

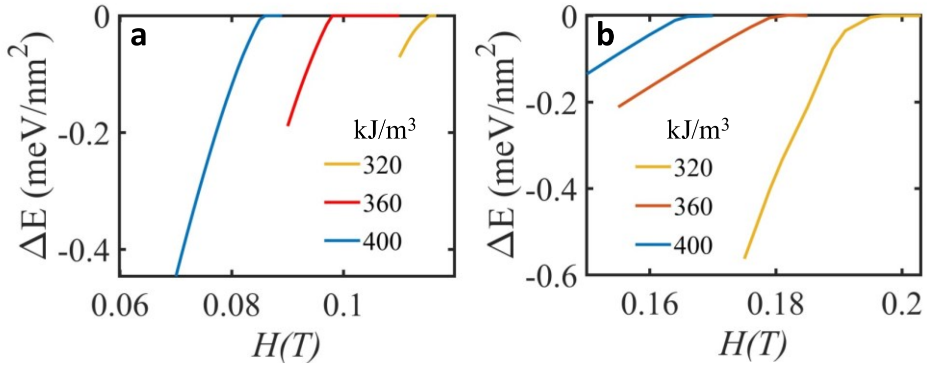

Figure S4.3. The energy difference between the maze and uniform states for 20 nm (a) and 30 nm (b).

Other than the skyrmions and maze domains, trivial bubbles can also be stabilized in our systems. Figure S4.4a shows stabilization of the trivial bubble state at same magnetic fields as skyrmions for Δx = +50% g-CoPt 20 nm. As the magnetic field increases, bubbles disappear at lower field strengths than skyrmions do, indicating reduced stability for bubbles and favoring the

formation of skyrmion states. Figure S4.4b displays the energies of the skyrmion and the bubble relative to the ferromagnetic state (energy set to zero). Consistent with expectations from Figure S4.4a, trivial bubbles exhibit a higher energy, implying a lower energy barrier compared to skyrmions.

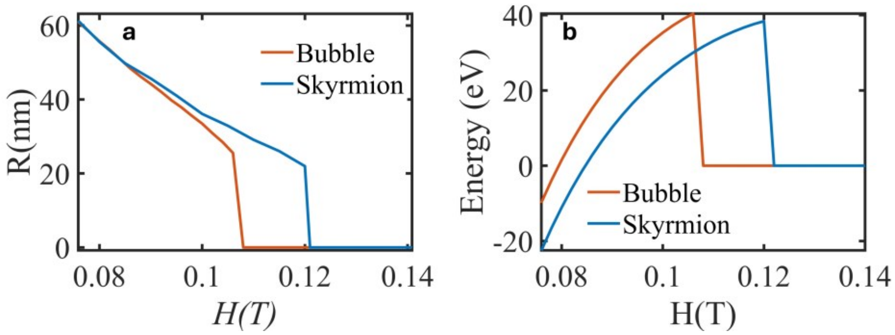

Figure S4.4. The radius of trivial bubble and skyrmion for Δx = +50% g-CoPt 20 nm (K_u =440 kJ/m^3) as applied field is increased (a) and energy of bubble and skyrmion shown in (b).

S5. Minimum Energy Path Calculations

In ferromagnetic films dipolar interactions may stabilize trivial magnetic bubbles or other textures such as antiskyrmions,[7] in addition to skyrmions. This makes it difficult to determine whether the solitons observed in the experiment are in fact skyrmions. To help support our conclusions we have carried out minimum energy path (MEP) calculations by the string method, using modified Mumax3 to examine the annihilation processes of skyrmions and trivial bubbles.[8] These calculations were used to find the least energy path for the decay of skyrmion and bubble initial states, as well as the energy barrier of the decay process. Since the rate of decay is given by $\Gamma = fe^{-\beta\Delta E}$ where ΔE is the energy barrier and f is the attempt frequency, this may provide some insight into the relative populations of these two types of solitons.[9]

The calculations were performed by relaxing an initial state of a skyrmion by the steepest conjugate gradient method, followed by running the MEP solver over 800 cycles, with 32 images in the transition chain. These calculations show that a skyrmion decaying to a uniform magnetic state could transition through a metastable trivial bubble state, before decaying to the ground state. Further, we see that the energy barrier for the transition from a trivial bubble to a skyrmion is much smaller than the reverse process. This suggests that while trivial bubbles may account for some fraction of the observed solitons in the sample, we can expect skyrmions to account for the majority.

Figure S5.1 shows the calculated energy and skyrmion winding number N_{sk} of the system as a function of the image number during the skyrmion decay for the Δx = +50% 20 nm g-CoPt film at a magnetic field of 85 mT. Here the initial state was chosen to be a skyrmion, while the final state is the homogeneous ground state. We see three energy minima in the plot, in order from left to right: trivial bubble, skyrmion, and homogeneous. The transition from the skyrmion to the bubble during the decay process does not require any initial input to the simulation; it occurs naturally during the decay of the skyrmion along the minimum energy path. The energy barriers for these parameters are much larger than room temperature, which suggests that bubbles and skyrmions are likely to coexist within the range of magnetic field where bubbles are metastable, as shown in figure S4.4. Given the magnitude of the energy difference between a skyrmion and a

bubble, as compared to the energy scale determined by temperature, we conclude that the appearance of bubbles is suppressed and may further lead to their decay into skyrmions.

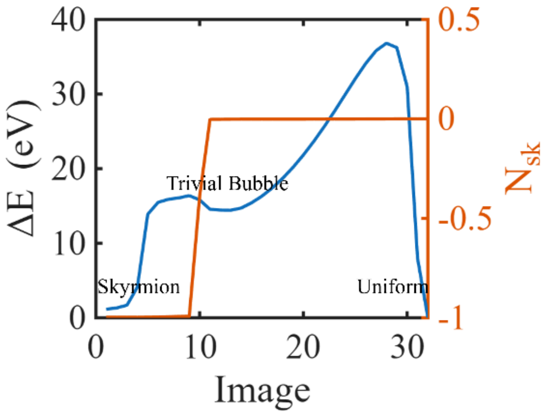

Figure S5.1. Energy during the transition from trivial bubble to uniform along the least energy path. Material parameters correspond to the 20 nm +50% gradient film, with an out of plane magnetic field of 85 mT. The lattice dimensions are 300 × 300 × 8 with a lattice constant of 2.5 nm. $K_u = 440\, kJ\, m^{-3}$, $D = 0.12\, mJ\, m^{-2}$. N_{sk} is the skyrmion winding number. The x-axis corresponds to the image number in the transition chain and can be interpreted as the reaction coordinate of the process.

We also compare the energy barrier for annihilation of a skyrmion for different magnetic fields, to the energy difference between the homogeneous state and skyrmion, shown in Figure S5.2. We can expect that when the energy difference between the skyrmion and the homogeneous state is positive, and the barrier for the transition is nonzero that skyrmions will be metastable states, with finite lifetimes. When the energy difference between the skyrmion and the homogeneous state is negative, it shows that homogeneous magnetization is no longer the ground state, and that the true ground state should be either maze domains or a skyrmion lattice. The point where the energy difference becomes negative should correspond roughly to the point where the skyrmion becomes unstable and is likely to expand into a maze.[10] Therefore, we should only expect to observe skyrmions within a finite range of magnetic fields, where the energy difference is positive, and the energy barrier is greater than zero.

Our calculations show qualitative agreement with the measurements presented in Figure 5b (main text), where skyrmions in the 20 nm positive gradient sample are only observed within the range of 150 to 210 mT (or 1.5 to 3 kOe). The calculations underestimate this range slightly; however this may be due to the lack of disorder, e.g., originating from nonuniformity of the sample. The inclusion of disorder would result in pinning which prevents the expansion of skyrmions at lower fields, and the decay of skyrmions at higher fields, thus affecting their stability.[11]

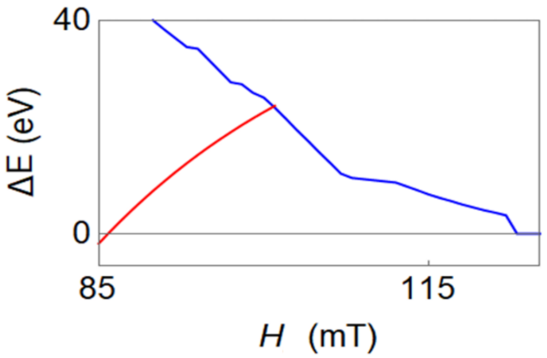

Figure S5.2. Energy barrier for transition from skyrmion to homogeneous (blue) compared to energy difference between skyrmion and homogeneous (red) for $\Delta x = +50\%$ 20 nm g-CoPt single layer. Results are obtained after 200 cycles with 30 images in the chain. $K_u = 440$ kJ/m^3, $D = 0.12$ mJ/m^2.

Figure S5.3 shows the simulated decay of skyrmions to homogeneous magnetization, for Δx = +50 20 nm g-CoPt film for several image snapshots. Skyrmion initially transitions to trivial bubble state, then the bubble gradually decreases in size until the uniform state is reached.

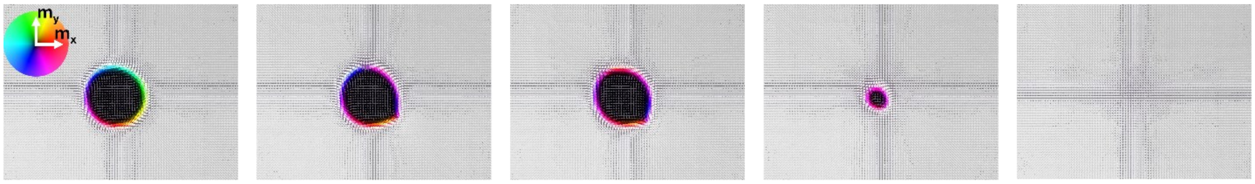

$N_{sk} = 1$ $N_{sk} = 1/2$ $N_{sk} = 0$ $N_{sk} = 0$ $N_{sk} = 0$

Figure S5.3. Images of a skyrmion to trivial bubble transmission and subsequent decay to uniform state done at *H* amplitude of 85 mT in +50% 20 nm g-CoPt film. White (Black) represents positive (negative) out-of-plane magnetization.

References:

[1] A. Erickson, Q. Zhang, H. Vakili, C. Li, S. Sarin, S. Lamichhane, L. Jia, I. Fescenko, E. Schwartz, S.-H. Liou, J. E. Shield, G. Chai, A. A. Kovalev, J. Chen, A. Laraoui, *ACS Nano* **2024**, *18*, 31261.

[2] J. Liang, M. Chshiev, A. Fert, H. Yang, *Nano Lett.* **2022**, *22*, 10128.

[3] Q. Zhang, J. Liang, K. Bi, L. Zhao, H. Bai, Q. Cui, H.-A. Zhou, H. Bai, H. Feng, W. Song, G. Chai, O. Gladii, H. Schultheiss, T. Zhu, J. Zhang, Y. Peng, H. Yang, W. Jiang, *Phys. Rev. Lett.* **2022**, *128*, 167202.

[4] G. Kimbell, C. Kim, W. Wu, M. Cuoco, J. W. A. Robinson, *Commun Mater* **2022**, *3*, 19.

[5] H. Vakili, Y. Xie, A. W. Ghosh, *Phys. Rev. B* **2020**, *102*, 174420.

[6] C.-E. Fillion, J. Fischer, R. Kumar, A. Fassatoui, S. Pizzini, L. Ranno, D. Ourdani, M. Belmeguenai, Y. Roussigné, S.-M. Chérif, S. Auffret, I. Joumard, O. Boulle, G. Gaudin, L. Buda-Prejbeanu, C. Baraduc, H. Béa, *Nat Commun* **2022**, *13*, 5257.

[7] A. A. Kovalev, S. Sandhoefner, *Front. Phys.* **2018**, *6*, 98.

[8] W. E, W. Ren, E. Vanden-Eijnden, *The Journal of Chemical Physics* **2007**, *126*, 164103.

[9] P. F. Bessarab, V. M. Uzdin, H. Jónsson, *Phys. Rev. B* **2012**, *85*, 184409.

[10] H. T. Diep, S. El Hog, A. Bailly-Reyre, *AIP Advances* **2018**, *8*, 055707.

[11] R. Gruber, J. Zázvorka, M. A. Brems, D. R. Rodrigues, T. Dohi, N. Kerber, B. Seng, M. Vafaee, K. Everschor-Sitte, P. Virnau, M. Kläui, *Nat Commun* **2022**, *13*, 3144.
